# Differences in Cardiorespiratory Fitness between Chinese and Japanese Children and Adolescents

**DOI:** 10.3390/ijerph16132316

**Published:** 2019-06-30

**Authors:** Xiaofang Yang, Xiaojian Yin, Liu Ji, Ge Song, Huipan Wu, Yuqiang Li, Guodong Wang, Cunjian Bi, Yi Sun, Ming Li, Ting Zhang, Hiroshi Kato, Akira Suzuki

**Affiliations:** 1 Key Laboratory of Adolescent Health Assessment and Exercise Intervention of Ministry of Education, East China Normal University, Shanghai 200241, China; 2 School of Physical Education & Health Care, East China Normal University, Shanghai 200241, China; 3 College of Economics and Management, Shanghai Institute of Technology, Shanghai 201418, China; 4 Department of Education, Wakayama University, Wakayama-city 640-8510, Japan; 5 Department of Sports Science, Daito Bunka University, Tokyo 355-8501, Japan

**Keywords:** children and adolescents, China and Japan, cardiorespiratory fitness, 20mSRT, socioeconomic status

## Abstract

Background: This study aimed to compare the difference in cardiorespiratory fitness between Chinese and Japanese children and adolescents. Methods: Participants comprised 9025 children and adolescents aged 7–18 years from China and Japan. Cardiorespiratory fitness (CRF) was measured by performance in the 20 m shuttle run test (20mSRT) and estimated maximal oxygen consumption (VO_2max_). Differences in CRF between countries were evaluated by *t*-tests. Centile curves for the 20mSRT and VO_2max_ values were constructed for Chinese and Japanese children and adolescents, respectively, using the Lambda Mu and Sigma (LMS) method. Results: (1) For most of the age groups, the 20mSRT and VO_2max_ performances among Chinese participants were lower than among Japanese participants. (2) Japanese children had the most apparent gains in *P*_10_, *P*_50_, and *P*_90_ VO_2max_ values in primary school; however, they gradually decreased in middle school. For Chinese girls, the *P*_10_, *P*_50_, and *P*_90_ VO_2max_ values decreased gradually with age. (3) The VO_2max_ value among Japanese children increased; however, it decreased or remained flat among Chinese children in primary school. Conclusions: CRF among Chinese participants was lower than among Japanese participants while the VO_2max_ value showed different trends in primary school. Effective measures should be taken to improve CRF among children and adolescents.

## 1. Introduction

Physical fitness is one of the important indices of health; it reflects not only the growth and development of the youth but also the national health level [1]. Cardiorespiratory fitness (CRF) is a health-related component of physical fitness, an independent determinant of health across lifespan that is associated with brain structure and function and decreased risk of all-cause mortality [2,3,4,5]. Numerous studies have identified that cardiorespiratory fitness in youth is negatively associated with cardiometabolic risk indicators, such as waist circumference, mean arterial blood pressure, fasting glucose, triglycerides, and high-density lipoprotein cholesterol [6,7]. In addition, longitudinal studies have found that low CRF in childhood and adolescence is related to increased cardiometabolic disease risk, BMI, body fat, metabolic syndrome, and early death in adulthood [8,9]. Therefore, the measurement and monitoring of CRF among children and adolescents is conducive to promoting the health status of the current and future population. The 20 m shuttle run test (20mSRT) is the most widely used field-based assessment of CRF, used in at least 50 countries [10]. Data on 1,142,026 children and adolescents aged 9–17 years from 50 countries have found variability among children and adolescents’ 20mSRT performance between regions. Children in North Central Europe and Africa have the highest cardiorespiratory fitness while those in South America have the lowest cardiorespiratory fitness. Among European countries, children in central-northern countries have better 20mSRT performance than their southern counterparts. Meanwhile, the variability between regions is relative to socioeconomic indicators (Gini index, human development index, and urbanization) and childhood obesity [11,12].

China and Japan are the second- and third-largest economies in the world, respectively, and the two largest economies in Asia. The United Nations Human Development Report showed that the human development index (HDI) value in China was less than Japan’s in 2011, and that China and Japan were a developing and a developed country, respectively [13]. Data from the World Bank in 2014 showed that the Gini index score in China was higher than that in Japan. Gini index is a measure of income inequality and a higher Gini index means more income difference between people [14]. Regarding urbanization, China has entered a period of rapid urbanization since reform and opening-up in 1978. The rate of urbanization increased from 19.39% to 51.27% between 1980 and 2011 [15]. Data from the World Bank showed that the proportion of urban population in China was less than Japan in 2014 [16]. Thus, socioeconomic indicators such as Gini index, human development index (HDI), and urbanization in China and Japan show differences. A recent study found that country-specific income inequality (Gini index) was a strong negative correlate of children’s 20mSRT performance [11]. Therefore, the potential influence of socioeconomic factors on the CRF between Chinese and Japanese children and adolescents needs to be further explored.

In recent years, several studies have noted changes in the CRF of Chinese and Japanese children and adolescents. The CRF of Chinese children and adolescents has gradually declined from 1985 to 2010 due to substantial changes in the society and lifestyle in China, such as more children being overweight and obese, more convenient transportation, sedentary behavior, and poor physical activity [17,18,19,20]. As for Japan, a report by the Ministry of Education, Culture, Sports, Science and Technology (MEXT) indicated that the performances in the 20 m shuttle run test (20mSRT) in 2014 were lower than those in the 1980s among children and adolescents [21]. Thus, it can be seen that CRF among children and adolescents in both China and Japan shows a decreasing trend over time; however, the relationship in terms of the CRF between Chinese and Japanese children and adolescents has not been revealed.

Consequently, the present study aimed to evaluate the CRF of children and adolescents aged 7–18 years in China and Japan using 20mSRT and estimated VO_2max_, with the aim of exploring the difference in CRF among children and adolescents between countries and with distinct socioeconomic contexts, and to analyze the causes of CRF difference, so as to provide a reference for promoting the physical health development of children and adolescents.

## 2. Materials and Methods

### 2.1. Participants and Sampling

In the spring of 2014, 9025 children and adolescents aged 7–18 years were chosen from Shanghai (urban and rural areas), Taiyuan (urban area), and Pinxingguan (rural area) of Shanxi province in China, and from Wakayama and Saitama in Japan using stratified random cluster sampling. With regard to the Chinese sampling, two provinces or municipalities were selected from eastern and northern China for the first stage of sampling (provincial level). For the second sampling stage, one provincial capital or municipality (urban area) and county or town (rural area) were selected in each of the provinces. The Chinese sample was 4558 (2277 boys, 2281 girls) and the Japanese sample was 4467 (2203 boys, 2264 girls), with valid data on weight, height, and CRF. Exclusion criteria for the participants were (1) reporting drug intake or a history of cancer, stroke, angina, heart failure, or myocardial infarction, and (2) having an athletic background or experience of competition in sports games. To ensure the effectiveness and comparability of the research, two typical provinces or municipalities were selected within the same latitudinal zone from both China and Japan for the sampling of participants. The climate types of all these cities are similar. The Chinese cities are located between 31.22 degrees north latitude (Shanghai) and 37.87 degrees north latitude (Taiyuan), and the Japanese cities are located between 34.23 degrees north latitude (Wakayama) and 35.86 degrees north latitude (Saitama). In the Chinese sample, the ratio of rural to urban sample size was 1:1. After 1946, Japan began to implement a process of urban–rural integration. By the 1990s, Japan had basically achieved the goal of narrowing the gap between urban and rural areas. Since reform and opening-up, China’s urban and rural development has been uneven, and the income gap between urban and rural residents has been widening [22,23]. Compared to China, little difference exists between urban and rural areas in Japan. Therefore, there was no emphasis on the factor of urban versus rural areas in the sampling of Japanese participants, but the sampling consideration of big versus small–medium cities was taken into account. Written informed consent from the parents or legal guardians and consent from each participant were obtained.

### 2.2. Anthropometric Measurements

The equipment for measuring height and weight were standardized. All tests were conducted according to a standardized program using the unified equipment at schools. All the participants were required to urinate fully and defecate before the test; boys wore only underwear, and girls wore a T-shirt and thin trousers. None of the participants wore shoes during the tests [24]. Height was recorded to the nearest 0.1 cm and measured against metal column scales, with knees not bent, arms at sides, shoulders relaxed, feet flat on the floor. Weight was measured using platform scales, and the results were recorded to the nearest 0.1 kg.

### 2.3. Evaluation Criteria for Nutrition

The present study was based on the 2007 WHO report on child growth standards, and examined the presence of underweight and overnutrition (overweight and obesity) among Chinese and Japanese children and adolescents [25]. Underweight, overweight, and obesity measurement adopted BMI-for-age screening (when BMI was minus 2Z-Score, we considered it underweight, above or equal to 1Z-Score was overweight, and above 2Z-Score was obese). BMI was calculated as weight (kg)/height (m)^2^.

### 2.4. Cardiorespiratory Fitness Test

In this study, CRF was estimated using the 20mSRT and estimated maximal oxygen consumption (VO_2max_). Participants ran back and forth on a 20 m course and were required to touch the 20 m line and, at the same time, a sound signal was emitted from a prerecorded tape. Participants started at a speed of 8.0 km/h, the second stage was at 9.0 km/h, and the speed was thereafter increased by 0.5 km/h each minute. The test was terminated when participants stopped due to fatigue, or when they failed to reach the end line concurrent with the signals on two consecutive occasions. The total laps completed was defined as the 20mSRT performance [26]. Tests were held in a gymnasium, playground, or hall with a nonslippery surface at schools; meanwhile, a variety of factors that may affect test results, such as rain, wind, and other environmental conditions, were strictly controlled. The equation of Matsuzaka et al. was used to evaluate the VO_2max_. To calculate the VO_2max_, the 20mSRT performance, gender, age, and BMI were entered into the formula: VO_2__max_ (mL·kg^−1^·min^−1^) = 61.1 − 2.20 (gender: M = 0; F = 1) − 0.462 (age) − 0.862 (BMI) + 0.192 (total laps) [27].

### 2.5. Ethical Consideration

The data for this research were approved by the Medical Ethics Committee of East China Normal University (ethically approved project identification code: HR2016/12055). All students’ names were digitally coded to avoid leaking of their personal information.

### 2.6. Statistical Analysis

The chi-square test was used to inspect the prevalence of underweight, overweight, and obesity. The *t*-test was used to evaluate the significant differences in 20mSRT and VO_2max_ performances between Chinese and Japanese children and adolescents. The 10th, 50th, and 90th percentiles of 20mSRT performance-for-age and VO_2max_ performance-for-age were computed using the Lambda Mu and Sigma (LMS) [28] method for Chinese and Japanese children and adolescents aged 7–18 years. The LMS method fits smooth centile curves to reference data by summarizing the changing distribution of three sex-specific and age-specific curves representing the skewness (L, expressed as a Box–Cox power), the median (M), and the coefficient of variation (S) [29]. The level of statistical significance was set at 0.05, and all analyses were conducted using the statistical software SPSS version 15.0 (IBM, Armonk, NY, USA).

## 3. Results

Table 1 shows that China has a higher Gini index, lower HDI, and lower proportion of urban population compared to Japan.

As shown in Table 2 and Table 3, the mean 20mSRT and predicted VO_2max_ performances of Chinese children and adolescents aged 7–18 were, in general, less than those of Japanese children and adolescents. Irrespective of sex, the 20m SRT and VO_2max_ performances among Chinese participants of most age groups were less than those of Japanese participants.

**Figure 1:**
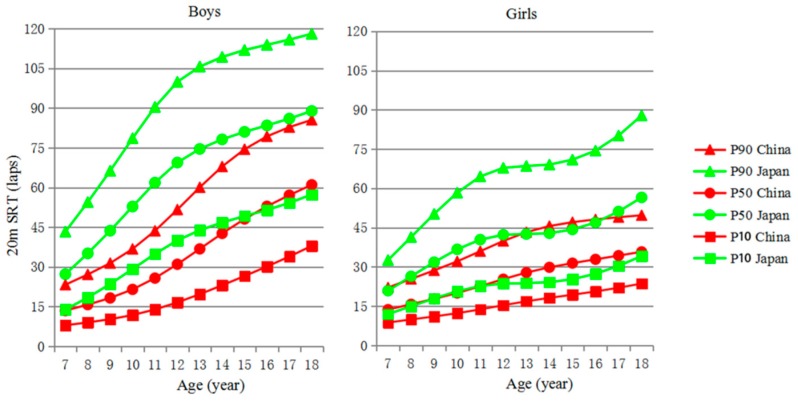
Centile curves for 20mSRT performances of Chinese and Japanese children and adolescents aged 7–18 years.

Centile curves for 20mSRT performance of Chinese and Japanese children and adolescents aged 7–18 years are given in Figure 1. The equivalent numerical values are available in Table 4. This figure contains curves approximating the 10th, 50th, and 90th centiles. Together, these data show that Japanese boys and girls in all age groups performed better on the test compared with their Chinese counterparts. The performances of Chinese and Japanese boys and girls improved with age.

**Figure 2:**
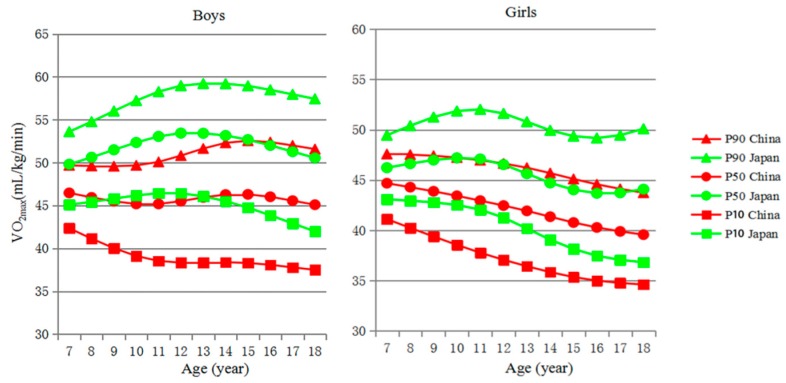
Centile curves for VO_2max_ values of Chinese and Japanese children and adolescents aged 7–18 years.

Figure 2 presents that the 10th, 50th and 90th percentiles of VO_2max_-for-age for Chinese children were greater compared to those for Japanese children. The equivalent numerical values are available in Table 5. Japanese boys had the most apparent gains in the 10th, 50th, and 90th percentiles between the ages of 7 and 12, and a gradual decrease from the age of 13. For Japanese girls, the *P*_50_ and *P*_90_ VO_2max_ value increased between the ages of 7 and 11. For Chinese girls aged 7–18, the *P*_10_, *P*_50_, and *P*_90_ VO_2max_ value decreased gradually with age. Meanwhile, Figure 2 also shows that the VO_2max_ value among Japanese children increased significantly, however, it decreased or remained flat among Chinese children in primary school.

Table 6 shows that the overall prevalence of overweight and obesity among Chinese participants was greater than Japanese participants (for boys: Chinese, 19.0% and 13.1%; Japanese, 11.0% and 4.3%, respectively. For girls: Chinese, 13.7% and 5.5%; Japanese, 8.5% and 1.2%, respectively). The prevalence of overweight and obesity among Chinese participants in most age groups was greater than that of Japanese participants.

## 4. Discussion

The present study demonstrated that the CRF of Chinese children and adolescents was less than that of Japanese participants, which may be related to socioeconomic status, BMI, physical activity status, and dietary habits among children and adolescents. Since reform and opening-up in 1978, increased income inequality has emerged in China. Data from the Chinese Health and Nutrition Survey (CHNS) in the period 1989 to 2011 showed that an increased Gini index was associated with higher risks of having abnormal obesity [30]. Among 205,388 girls aged 13–14 years from 36 countries, those from higher income inequality countries had a greater median BMI [31]. BMI was closely correlated to CRF among children and adolescents. The relationship between BMI and CRF among children was parabolic, and the poorest CRF occurred with the highest BMI, while the best CRF occurred with normal weight [32,33]. In addition, overweight and obesity were significantly negatively correlated with CRF in children and adolescents [34], and obesity led to a decrease in children’s CRF [35]. Meanwhile, the results of the present study showed that the prevalence of overweight and obesity among Chinese participants in most age groups was greater, and the prevalence of normal weight was lower than that of Japanese participants. Therefore, the difference in BMI among Chinese and Japanese participants may be related to a significantly lower CRF among children in China than in Japan. Regarding the physical activity status, a survey of the mode of commuting to school among children from grade 4 to grade 12 in China found that among primary school, middle school, and high school students, the proportion taking an active mode of commuting (cycling or walking) to school was 55.8%, 54.6%, and 50.9%, respectively [36]. The 2015 Sasakawa Sports Foundation (SSF) National Sports-Life Survey of Young People reported that 93% of Japanese elementary school children regularly commute actively to school, while 88% of those at junior high school and 68% of senior high school students regularly commute actively to school [37]. Thus, it can be seen that the proportion of cycling and walking to school among Chinese children was lower than Japanese children. Among children, the mode of commuting to school is related to CRF [38,39,40]. Among Colombian children and adolescents aged 9–18 years, cycling to school was found to be associated with greater CRF [38]. Likewise, in English schoolchildren aged 10–16 years, there was a positive association between walking and cycling to school and CRF [39]. Among Chinese Han children aged 10–18 years, walking and cycling to school was also found to be significantly associated with greater CRF in girls [40]. Therefore, physical activity status could be an important reason for the CRF of Chinese participants being significantly lower than that of Japanese participants. With the development of economy and urbanization, China has been undergoing a nutrition transition to a westernized diet characterized by low grain intake and a high consumption of edible oils and animal-sourced foods [41]. Data from the Chinese Health and Nutrition Survey (CHNS) regarding children aged 12–17 years showed a structural change in dietary habits from carbohydrates to fat and protein from 1991 to 2011 [42]. Additionally, a recent study found that there were many problems in dietary nutrition for children and adolescents in China, such as an unreasonable nutritional allocation among three meals per day, a greater proportion of children who skip breakfast, and the intake of high-fat and high-salt foods [43]. The CRF of children and adolescents may indeed be affected by dietary habits. For English boys aged 10–16 years, regularly eating breakfast during school days was positively associated with CRF [44]. Among French primary school children, the frequency of breakfast was positively associated with CRF, while a cumulation of unhealthy eating habits was negatively associated with CRF [45]. Therefore, dietary factors may be associated with the differences observed in CRF between Chinese and Japanese participants.

The present study also found that the VO_2max_ value among Japanese children increased significantly; however, it decreased or remained flat among Chinese children in primary school, which could be related to physical activity status, dietary nutrition, and sleep duration. As mentioned previously, the proportion of active travel to school among Chinese and Japanese elementary school children was 55.8% and 93%, respectively [36,37]. Active travel to school was associated with greater CRF among children. Therefore, physical activity status may lead to the different trend of VO_2max_ value among Chinese and Japanese children in primary school. Regarding dietary nutrition, the Japanese government enacted the School Lunch Program Law in 1954, and almost all the elementary schools in Japan enforced the school lunch program in 2013. The school lunch program has contributed to an improvement in nutrient intake and the establishment of appropriate dietary habits among elementary school students [46]. With the rapid development of China’s economy, great changes have taken place in people’s eating behaviors and lifestyles. Unreasonable breakfast structure and snacking was popular, and the amount of beverage intake had increased among Chinese primary school students over the past decade [47,48]. As mentioned previously, unhealthy eating habits were negatively related to CRF among primary school students. Therefore, dietary nutrition may lead to the VO_2max_ value among Chinese and Japanese children in the elementary school showing different trends. Regarding sleep duration, insufficient sleep duration (<9 h/d) led to decreased CRF among children [49]. Data from the 2014 Chinese National Survey on Students Constitution and Health showed that the prevalence of insufficient sleep for primary school children was 66.6% [50]. Meanwhile, a recent study found the prevalence of insufficient sleep for Japanese primary school children was 24.4% [51]. Therefore, sleep duration may lead to different trends in the VO_2max_ values among Chinese and Japanese primary school children.

## 5. Conclusions

The present study investigated and analyzed the CRF of Chinese and Japanese children and adolescents, so as to provide a theoretical and practical basis for improving the health level of children and adolescents. Findings from this study confirm that CRF among Chinese children was less than among Japanese children. Meanwhile, the VO_2max_ value among Japanese children increased significantly, however, the value decreased or remained flat among Chinese children in primary school. Based on these results, policymakers, schools, and families should work together to strengthen health education, develop healthy dietary habits and sufficient sleep duration, control weight, reduce sedentary behavior, and increase the physical activity levels among children and adolescents, especially primary school children. There are also some study limitations to note. First, the present study has not investigated children and adolescents’ lifestyles, such as physical activity and dietary habits. Second, this study is cross-sectional and unable to determine causality. Therefore, cohort studies and lifestyle surveys should be conducted among Chinese and Japanese children and adolescents in the future. In addition, effective strategies should be taken to control the weight of children and adolescents, to minimize childhood overweight and obesity and to improve CRF.

## Figures and Tables

**Table 1:** The descriptive socioeconomic indicators of China and Japan.

Year	Country	Human Development Index (HDI) ^a^	Gini Index ^b^	Urban Population ^c^	Urban Population ^d^ (% of total) ^d^	Urban Population Growth (annual %) ^e^
2000	China	0.588	-	452,999.15	36	3.6
	Japan	0.868	-	99,760.75	79	0.3
2008	China	-	42.9	616,481.19	47	3.4
	Japan	-	32.1	114,107.98	89	1.1
2010	China	0.682	-	658,498.66	49	3.3
	Japan	0.899	-	116,302.93	91	0.9
2014	China	-	-	740,239.26	54	2.8
	Japan	-	-	116,208.08	91	0.0

**^a^** HDI from UNDP Human Development Reports 2011 (2008 and 2014 data not available); **^b^** Gini Index: the World Bank http://data.worldbank.org/indicator/SI.POV.GINI/countries?display=default (2000, 2010, and 2014 data not available); ^**c**,**d**,**e**^ World Bank http://data.worldbank.org/indicator/SP.URB.TOTL.IN.ZS?display=default.

**Table 2:** Differences in 20 m shuttle run test (20mSRT) performance between Chinese and Japanese children and adolescents (laps).

Age (Year)	*N*1, *N*2	Boy		*N*1, *N*2	Girl	
		China	Japan		China	Japan
7	192, 191	14.73 ± 5.51*	29.08 ± 12.45	190, 192	14.66 ± 5.39 *	21.96 ± 7.50
8	190, 190	17.68 ± 8.26 *	33.39 ± 13.34	189, 191	17.03 ± 6.69 *	25.95 ± 11.49
9	189, 188	19.52 ± 9.27 *	43.77 ± 16.47	188, 192	19.68 ± 7.78 *	32.89 ± 13.01
10	194, 192	22.41 ± 10.53 *	50.93 ± 17.34	191, 190	19.34 ± 7.70 *	38.35 ± 13.68
11	189, 178	26.08 ± 11.14 *	62.44 ± 19.40	189, 192	23.90 ± 9.26 *	46.02 ± 16.84
12	191, 170	32.61 ± 14.54 *	87.10 ± 24.00	190, 191	26.32 ± 10.11 *	54.25 ± 13.72
13	191, 191	38.81 ± 15.85 *	70.86 ± 19.22	192, 194	32.55 ± 11.10 *	41.24 ± 14.34
14	187, 193	46.34 ± 19.03 *	76.70 ± 22.14	188, 193	32.06 ± 11.03 *	36.09 ± 20.30
15	187, 166	52.58 ± 19.29 *	83.91 ± 25.89	194, 171	30.90 ± 11.49 *	52.88 ± 20.85
16	193, 179	56.43 ± 20.31 *	77.57 ± 25.90	188, 188	33.55 ± 10.87 *	44.05 ± 13.13
17	188, 180	57.63 ± 17.72 *	80.91 ± 25.63	190, 187	34.45 ± 10.73 *	46.23 ± 13.46
18	186, 185	59.20 ± 17.55 *	91.71 ± 21.72	192, 183	36.69 ± 9.38*	70.13 ± 22.40
Total	2277, 2203	36.91 ± 21.95 *	65.14 ± 29.18	2281, 2264	26.78 ± 11.95 *	42.29 ± 19.90

Note: (1) N1 = China, N2 = Japan; (2) *t*-test; * *p* < 0.05.

**Table 3:** Differences in estimated maximal oxygen consumption (VO_2max_) performance between Chinese and Japanese children and adolescents (mL/kg/min).

Age (Year)	*N*1, *N*2	Boy		*N*1, *N*2	Girl	
		China	Japan		China	Japan
7	192, 191	46.26 ± 3.36 *	49.74 ± 3.39	190, 192	44.56 ± 2.41 *	46.36 ± 2.16
8	190, 190	45.39 ± 4.03 *	49.77 ± 3.71	189, 191	43.82 ± 3.31 *	46.34 ± 2.97
9	189, 188	45.25 ± 3.75 *	51.09 ± 4.26	188, 192	43.83 ± 3.19 *	46.81 ± 3.66
10	194, 192	44.41 ± 4.41 *	51.46 ± 4.99	191, 190	42.95 ± 3.71 *	47.21 ± 3.76
11	189, 178	44.41 ± 4.42 *	52.74 ± 5.04	189, 192	42.59 ± 3.31 *	47.62 ± 4.72
12	191, 170	44.87 ± 5.25 *	55.12 ± 4.41	190, 191	41.75 ± 4.28 *	48.08 ± 3.42
13	191, 191	45.27 ± 5.29 *	51.72 ± 4.77	192, 194	42.04 ± 3.96 *	45.06 ± 3.20
14	187, 193	45.78 ± 6.32 *	52.84 ± 5.15	188, 193	41.24 ± 3.82 *	43.48 ± 4.11
15	187, 166	45.73 ± 5.93 *	53.02 ± 5.87	194, 171	39.59 ± 4.28 *	44.19 ± 5.04
16	193, 179	45.45 ± 6.23 *	50.76 ± 5.57	188, 188	39.87 ± 3.84 *	42.19 ± 3.06
17	188, 180	44.94 ± 5.19 *	50.69 ± 5.53	190, 187	39.43 ± 3.64 *	41.69 ± 3.27
18	186, 185	45.07 ± 5.14 *	51.80 ± 5.60	192, 183	39.82 ± 3.41 *	46.50 ± 5.08
Total	2277, 2203	45.23 ± 5.04 *	51.69 ± 5.09	2281, 2264	41.78 ± 4.02 *	45.48 ± 4.29

Note: (1) N1 = China, N2 = Japan; (2) *t*-test; * *p* < 0.05.

**Table 4:** The 20-metre shuttle run (number of laps) centiles by age and sex in Chinese and Japanese children and adolescents aged 7–18 years.

Sex	Age (Year)	L	M	S	China			L	M	S	Japan		
					*P*_10_	*P*_50_	*P*_90_				*P*_10_	*P*_50_	*P*_90_
Boy	7	−0.05	13.51	0.42	7.95	13.51	23.29	0.67	27.28	0.42	13.93	27.28	43.32
	8	0.03	15.76	0.43	9.06	15.76	27.17	0.72	35.12	0.40	18.43	35.12	54.50
	9	0.11	18.28	0.44	10.26	18.28	31.44	0.77	43.69	0.38	23.55	43.69	66.32
	10	0.19	21.52	0.44	11.82	21.52	36.80	0.82	52.83	0.37	29.20	52.83	78.61
	11	0.28	25.79	0.44	13.94	25.79	43.63	0.87	61.85	0.35	34.95	61.85	90.42
	12	0.36	31.02	0.44	16.61	31.02	51.67	0.92	69.45	0.34	40.03	69.45	99.92
	13	0.44	36.82	0.43	19.71	36.82	60.08	0.97	74.58	0.32	43.86	74.58	105.67
	14	0.52	42.67	0.41	23.07	42.67	67.94	1.02	78.20	0.31	46.79	78.20	109.32
	15	0.60	48.12	0.39	26.57	48.12	74.46	1.07	81.04	0.30	49.22	81.04	111.95
	16	0.68	52.92	0.36	30.17	52.92	79.34	1.12	83.46	0.29	51.53	83.46	113.91
	17	0.77	57.13	0.34	33.93	57.13	82.83	1.18	86.04	0.28	54.20	86.04	115.88
	18	0.85	61.05	0.31	37.97	61.05	85.57	1.23	88.92	0.26	57.36	88.92	118.08
Girl	7	−0.14	13.67	0.36	8.73	13.67	22.10	0.47	20.91	0.38	11.95	20.91	32.56
	8	−0.08	15.71	0.37	9.89	15.71	25.36	0.45	26.34	0.39	14.94	26.34	41.35
	9	−0.01	17.77	0.37	11.04	17.77	28.64	0.44	31.78	0.40	17.94	31.78	50.21
	10	0.06	20.00	0.37	12.28	20.00	32.08	0.42	36.69	0.40	20.65	36.69	58.31
	11	0.13	22.58	0.37	13.75	22.58	35.96	0.40	40.39	0.40	22.67	40.39	64.52
	12	0.20	25.30	0.37	15.33	25.30	39.87	0.39	42.25	0.41	23.67	42.25	67.81
	13	0.27	27.81	0.37	16.84	27.81	43.24	0.37	42.53	0.41	23.83	42.53	68.53
	14	0.34	29.82	0.36	18.15	29.82	45.58	0.35	42.83	0.41	24.11	42.83	69.05
	15	0.41	31.42	0.34	19.34	31.42	47.07	0.34	44.28	0.40	25.29	44.28	70.94
	16	0.48	32.87	0.33	20.60	32.87	48.12	0.32	46.94	0.39	27.35	46.94	74.39
	17	0.55	34.30	0.31	22.02	34.30	48.95	0.30	51.12	0.38	30.36	51.12	80.16
	18	0.62	35.74	0.29	23.60	35.74	49.70	0.29	56.52	0.37	34.12	56.52	87.82

Note: L, skew; M, median; S, coefficient of variation; *P*, percentile.

**Table 5:** Relative maximal oxygen uptake (VO_2max_, mL/kg/min) centiles by age and sex in Chinese and Japanese children and adolescents aged 7–18 years.

Sex	Age (Year)	L	M	S	China			L	M	S	Japan		
					*P*_10_	*P*_50_	*P*_90_				*P*_10_	*P*_50_	*P*_90_
Boy	7	4.15	46.47	0.06	42.35	46.47	49.68	3.52	49.80	0.07	45.09	49.80	53.60
	8	3.95	45.94	0.07	41.14	45.94	49.60	3.42	50.62	0.07	45.42	50.62	54.77
	9	3.76	45.49	0.08	40.03	45.49	49.58	3.32	51.49	0.08	45.80	51.49	56.01
	10	3.56	45.18	0.09	39.11	45.18	49.67	3.22	52.35	0.08	46.17	52.35	57.23
	11	3.37	45.18	0.10	38.54	45.18	50.08	3.12	53.05	0.09	46.43	53.05	58.27
	12	3.17	45.50	0.10	38.34	45.50	50.82	3.02	53.44	0.09	46.42	53.44	58.96
	13	2.98	45.94	0.11	38.34	45.94	51.64	2.92	53.42	0.09	46.07	53.42	59.21
	14	2.78	46.28	0.11	38.38	46.28	52.30	2.83	53.16	0.10	45.50	53.16	59.20
	15	2.59	46.30	0.12	38.30	46.30	52.55	2.73	52.68	0.10	44.75	52.68	58.95
	16	2.39	46.02	0.12	38.08	46.02	52.39	2.63	52.01	0.11	43.86	52.01	58.48
	17	2.20	45.56	0.12	37.79	45.56	52.00	2.53	51.28	0.11	42.92	51.28	57.95
	18	2.00	45.09	0.12	37.50	45.09	51.58	2.43	50.55	0.12	41.98	50.55	57.43
Girl	7	3.86	44.68	0.06	41.12	44.68	47.58	0.58	46.22	0.05	43.09	46.22	49.45
	8	3.74	44.29	0.06	40.24	44.29	47.52	0.73	46.63	0.06	42.94	46.63	50.40
	9	3.61	43.88	0.07	39.39	43.88	47.41	0.88	46.99	0.07	42.78	46.99	51.25
	10	3.49	43.43	0.08	38.56	43.43	47.23	1.03	47.20	0.08	42.53	47.20	51.86
	11	3.37	42.95	0.08	37.78	42.95	46.97	1.18	47.09	0.08	42.06	47.09	52.02
	12	3.25	42.46	0.09	37.07	42.46	46.64	1.33	46.54	0.09	41.26	46.54	51.62
	13	3.12	41.94	0.09	36.43	41.94	46.22	1.48	45.64	0.09	40.18	45.64	50.79
	14	3.00	41.35	0.09	35.85	41.35	45.69	1.63	44.71	0.09	39.07	44.71	49.94
	15	2.88	40.78	0.09	35.35	40.78	45.10	1.79	44.04	0.10	38.15	44.04	49.36
	16	2.76	40.29	0.09	35.00	40.29	44.57	1.94	43.69	0.10	37.47	43.69	49.17
	17	2.63	39.89	0.09	34.77	39.89	44.12	2.09	43.74	0.11	37.06	43.74	49.45
	18	2.51	39.57	0.09	34.62	39.57	43.73	2.24	44.08	0.12	36.83	44.08	50.09

Note: L, skew; M, median; S, coefficient of variation; *P*, percentile.

**Table 6:** Rate of underweight, overweight, and obesity between Chinese and Japanese children and adolescents (N (%)).

Age (Year)	Country	Boy						Girl					
		N	Underweight	Normal Weight	Overweight	Obese	*p* value	N	Underweight	Normal Weight	Overweight	Obese	*p* value
7	China	192	6 (3.1)	129 (67.2)	27 (14.1)	30 (15.6)	0.002	190	3 (1.6)	146 (76.8)	26 (13.7)	15 (7.9)	0.001
	Japan	191	5 (2.6)	158 (82.7)	18 (9.4)	10 (5.2)		192	0 (0.0)	170 (88.5)	20 (10.4)	2 (1.0)	
8	China	190	3 (1.6)	107 (56.3)	40 (21.1)	40 (21.1)	<0.001	189	9 (4.8)	123 (65.1)	35 (18.5)	22 (11.6)	<0.001
	Japan	190	3 (1.6)	153 (80.5)	21 (11.1)	13 (6.8)		191	3 (1.6)	160 (83.8)	24 (12.6)	4 (2.1)	
9	China	189	9 (4.8)	104 (55.0)	38 (20.1)	38 (20.1)	<0.001	188	12 (6.4)	130 (69.1)	34 (18.1)	12 (6.4)	0.015
	Japan	188	0 (0.0)	159 (84.6)	17 (9.0)	12 (6.4)		192	4 (2.1)	158 (82.3)	24 (12.5)	6 (3.1)	
10	China	194	4 (2.1)	106 (54.6)	49 (25.3)	35 (18.0)	<0.001	191	18 (9.4)	132 (69.1)	29 (15.2)	12 (6.3)	<0.001
	Japan	192	4 (2.1)	156 (81.3)	20 (10.4)	12 (6.3)		190	2 (1.1)	167 (87.9)	15 (7.9)	6 (3.2)	
11	China	189	5 (2.6)	107 (56.6)	47 (24.9)	30 (15.9)	<0.001	189	2 (1.1)	143 (75.7)	33 (17.5)	11 (5.8)	0.032
	Japan	178	2 (1.1)	145 (81.5)	22 (12.4)	9 (5.1)		192	8 (4.2)	156 (81.3)	24 (12.5)	4 (2.1)	
12	China	191	4 (2.1)	127 (66.5)	35 (18.3)	25 (13.1)	<0.001	190	12(6.3)	131 (68.9)	32 (16.8)	15 (7.9)	<0.001
	Japan	170	3 (1.8)	114 (67.1)	38 (22.3)	15 (8.8)		191	8 (4.2)	173 (90.6)	10 (5.2)	0 (0.0)	
13	China	191	7 (3.7)	125 (65.4)	36 (18.8)	23 (12.0)	<0.001	192	5 (2.6)	146 (76.0)	30 (15.6)	11 (5.7)	<0.001
	Japan	191	5 (2.6)	160 (83.8)	21 (11.0)	5 (2.6)		194	6 (3.1)	183 (94.3)	4 (2.1)	1 (0.5)	
14	China	187	8 (4.3)	128 (68.4)	33 (17.6)	18(9.6)	<0.001	188	7 (3.7)	157 (83.5)	16 (8.5)	8 (4.3)	0.224
	Japan	193	4 (2.1)	173 (89.6)	14 (7.3)	2 (1.0)		193	7 (3.6)	173 (89.6)	10 (5.2)	3 (1.6)	
15	China	187	11 (5.9)	119 (63.6)	39 (20.9)	18 (9.6)	0.001	194	10 (5.2)	146 (75.3)	28 (14.4)	10 (5.2)	0.009
	Japan	166	9 (5.4)	135 (81.3)	17 (10.2)	5 (3.0)		171	2 (1.2)	143 (83.6)	25 (14.6)	1 (0.6)	
16	China	193	2 (1.0)	143 (74.1)	28 (14.5)	20 (10.4)	0.001	188	5 (2.7)	161 (85.6)	18 (9.6)	4 (2.1)	0.101
	Japan	179	6 (3.4)	150 (83.8)	20 (11.2)	3 (1.7)		188	4 (2.1)	173 (92.0)	11 (5.9)	0 (0.0)	
17	China	188	3 (1.6)	136 (72.3)	37 (19.7)	12 (6.4)	<0.001	190	1 (0.5)	167 (87.9)	21 (11.1)	1 (0.5)	0.568
	Japan	180	4 (2.2)	160 (88.9)	14 (7.8)	2 (1.1)		187	1 (0.5)	171 (91.4)	15 (8.0)	0 (0.0)	
18	China	186	14 (7.5)	139 (74.7)	24 (12.9)	9 (4.8)	0.488	192	3 (1.6)	174 (90.6)	11 (5.7)	4 (2.1)	0.517
	Japan	185	8 (4.3)	149 (80.5)	21 (11.4)	7 (3.8)		183	5 (2.7)	167 (91.3)	10 (5.5)	1(0.5)	
Total	China	2277	76 (3.3)	1470 (64.6)	433 (19.0)	298 (13.1)	<0.001	2281	87(3.8)	1756 (77.0)	313 (13.7)	125 (5.5)	<0.001
	Japan	2203	53 (2.4)	1812 (82.3)	243 (11.0)	95 (4.3)		2264	50 (2.2)	1994 (88.1)	192 (8.5)	28 (1.2)	

Note: The statistical analysis is χ^2^-test.

## References

[1] Yang H. (2011). Deepening sunshine sports project and promoting adolescent’s fitness. J. Beijing Sport Univ..

[2] Lee D.C., Artero E.G., Sui X.M., Blair S.N. (2010). Mortality trends in the general population: The importance of cardiorespiratory fitness. J. Psychopharmacol..

[3] Ferreira I., Twisk J.W., Mechelen W., Kemper H.C., Stehouwer C.D. (2005). Development of fatness, fitness, and lifestyle from adolescence to the age of 36 years: Determinants of the metabolic syndrome in young adults: The Amsterdam growth and health longitudinal study. Arch. Int. Med..

[4] Pontifex M.B., Hillman C., Kramer A.F., Chaddock L., Voss M.W., Cohen N.J., Kramer A.F., Hillman C.H. (2011). Cardiorespiratory fitness and the flexible modulation of cognitive control in preadolescent children. J. Cogn. Neurosci.

[5] Shah R.V., Murthy V.L., Colangelo L.A., Reis J., Venkatesh B.A., Sharma R., Abbasi S.A., Goff D.C.Jr., Carr J.J., Rana J.S. (2016). Association of fitness in young adulthood with survival and cardiovascular risk: The coronary artery risk development in young adults (CARDIA) study. JAMA Int. Med..

[6] Dos Santos F.K., Prista A., Gomes T.N.Q.F., Santos D., Damasceno A., Madeira A., Katzmarzyk P.T., Maia J.A.R. (2015). Body mass index, cardiorespiratory fitness and cardiometabolic risk factors in youth from Portugal and Mozambique. Int. J. Obes..

[7] Steele R.M., Brage S., Corder K., Wareham N.J., Ekelund U. (2008). Physical activity, cardiorespiratory fitness, and the metabolic syndrome in youth. J. Appl. Physiol..

[8] Mintjens S., Menting M.D., Daams J.G., Van Poppel M.N.M., Roseboom T.J., Gemke R.J.B.J. (2018). Cardiorespiratory Fitness in Childhood and Adolescence Affects Future Cardiovascular Risk Factors: A Systematic Review of Longitudinal Studies. Sports Med..

[9] Högström G., Nordström A., Nordström P. (2016). Aerobic fitness in late adolescence and the risk of early death: A prospective cohort study of 1.3 million Swedish men. Int. J. Epidemiol..

[10] Tomkinson G.R., Lang J.J., Tremblay M.S., Dale M., LeBlanc A.G., Belanger K., Ortega F.B., Léger L. (2017). International normative 20 m shuttle run values from 1,142,026 children and youth representing 50 countries. Br. J. Sports Med..

[11] Lang J.J., Tremblay M.S., Léger L., Olds T., Tomkinson G.R. (2018). International variability in 20 m shuttle run performance in children and youth: Who are the fittest from a 50-country comparison? A systematic literature review with pooling of aggregate results. Br. J. Sports Med..

[12] Ortega F.B., Ruiz J.R., Labayen I., Martínez-Gómez D., Vicente-Rodriguez G., Cuenca-García M., Gracia-Marco L., Manios Y., Beghin L., Molnar D. (2014). Health Inequalities in Urban Adolescents: Role of Physical Activity, Diet, and Genetics. Pediatrics.

[13] United Nations Development Programme (2011). Human Development Reports. http://www.undp.org/content/dam/undp/library/corporate/HDR/2011%20Global%20HDR/English/HDR_2011_EN_Complete.pdf.

[14] The World Bank Gini index (World Bank estimate). http://data.worldbank.org/indicator/SI.POV.GINI/countries?display=default.

[15] Wang Q. (2014). Effects of urbanisation on energy consumption in China. Energy Policy.

[16] The World Bank Urban population (% of total). http://data.worldbank.org/indicator/SP.URB.TOTL.INZS?display=default.

[17] Li H., Zhang Y., Ji C. (2012). Secular change of cardiorespiratory fitness in Chinese children and adolescents: 1985-2010. J. Sci. Med. Sport.

[18] Sun H., Ma Y., Han D., Pan C.W., Xu Y. (2014). Prevalence and trends in obesity among China’s children and adolescents, 1985–2010. PLoS ONE.

[19] Ren H., Zhou Z., Liu W.K., Wang X., Yin Z. (2017). Excessive homework, inadequate sleep, physical inactivity and screen viewing time are major contributors to high paediatric obesity. Acta Paediatr..

[20] Mak K.K., Ho S.Y., Lo W.S., McManus A.M., Lam T.H. (2011). Prevalence of exercise and non-exercise physical activity in Chinese adolescents. Int. J. Behav. Nutr. Phys. Act..

[21] Ministry of Education Culture, Sports, Science and Technology. The Report of Survey on Physical Strength and Athletic Performance. http://www.mext.go.jp/a_menu/sports/kodomo/zencyo/1342657.htm.

[22] Kong X.L. (2008). Evolution and enlightenment of Japanese urban and rural integration after the war. Expand. Horiz..

[23] Liu Z.Y. (2006). Study on the Status, Venture and measure of the gap between the urban and the rural in China. J. Huazhong Univ. Sci. Technol. (Soc. Sci. Ed.).

[24] Ji C.Y., Chen T.J., Sun X. (2013). Secular changes on the distribution of body mass index among Chinese children and adolescents, 1985–2010. Biomed. Env. Sci..

[25] WHO Child Growth Standards, Genava World Health Organisation. http://www.who.int/growthref/en/.

[26] Cooper Institute for Aerobics Research (1992). The Prudential FITNESSGRAM Test Administration Manual.

[27] Matsuzaka A., Takahashi Y., Yamazoe M., Kumakura N., Ikeda A., Wilk B., Bar-Or O. (2004). Validity of the multistage 20-m shuttle run test for Japanese children adolescents and adults. Pediatr. Exerc. Sci..

[28] Cole T.J., Green P.J. (1992). Smoothing reference centile curves: the LMS method and penalized likelihood. Stat. Med..

[29] Pan H., Cole T. (2010). User’s Guide to LMS Chartmaker.

[30] Bakkeli N.Z. (2016). Income inequality and health in China: A panel data analysis. Soc. Sci. Med..

[31] Murphy R., Stewart A.W., Hancox R.J., Wall C.R., Braithwaite I., Beasley R., Mitchell E.A., ISAAC Phase Three Study Group (2018). Obesity, underweight and BMI distribution characteristics of children by gross national income and income inequality: Results from an international survey. Obes. Sci. Pr..

[32] Li M., Yin X.J., Li Y.Q., Chai X.J., Ren S.E., Liu Y., Ling M.M. (2017). Correlation between BMI and 20mSRT in Children and Adolescents. Chin. J. Sch. Health.

[33] Al-Asiri Z.A., Shaheen A.A.M. (2015). Body mass index and health related physical fitness in Saudi girls and adolescents aged 8-15 years. Open J. Reha.

[34] Shang X.W., Liu A.L., Li Y.P., Hu X.Q., Du L., Ma J., Xu G.F., Li Y., Guo H.W., Ma G.S. (2010). The association of weight status with physical fitness among Chinese children. Int. J. Pediatr..

[35] Cruz A.G., Suárez J.F., Ciro J.O., Rodríguez C.N., Gallo V.J. (2014). Association between nutritional status and physical abilities in children aged 6-18 years in Medellin (Colombia). Pediatr. (Barc).

[36] Sun Y., Liu Y., Tao F.B. (2015). Associations Between Active Commuting to School, Body Fat, and Mental Well-being: Population-Based, Cross-Sectional Study in China. J. Adolesc Health.

[37] Tanaka C., Tanaka S., Inoue S., Miyachi M., Suzuki K., Reilly J.J. (2016). Results from Japan’s 2016 Report Card on Physical Activity for Children and Youth. J. Phys. Act. Health.

[38] Ramírez-Vélez R., García-Hermoso A., Agostinis-Sobrinho C., Mota J., Santos R., Correa-Bautista J.E., Amaya-Tambo D.C., Villa-González E. (2017). Cycling to school and body composition, physical fitness, and metabolic syndrome in children and adolescents. J. Pediatr..

[39] Voss C., Sandercock G. (2010). Aerobic fitness and mode of travel to school in English schoolchildren. Med. Sci. Sports Exerc..

[40] Yang X.F., Yin X.J., Li Y.Q., Chai X.J., Ren S.E., Liu Y., Ling M.M. (2017). Associations between physical activity, screen time and 20-meter shuttle run test performances among Chinese Han children and adolescents. Chin. J. Sch. Health.

[41] Cui Z., Dibley M.J. (2012). Trends in dietary energy, fat, carbohydrate and protein intake in Chinese children and adolescents from 1991 to 2009. Br. J. Nutr..

[42] Yu A.Y.L., López-Olmedo N., Popkin B.M. (2018). Analysis of dietary trends in Chinese adolescents from 1991 to 2011. Asia Pac. J. Clin. Nutr..

[43] Cai J.Y., Wang F., Liu X.X., Dai T. (2013). International experience and revelation of the child nutrition improvement measures. Chin. J. Health Educ..

[44] Sandercock G.R.H., Voss C., Dye L. (2010). Associations between habitual school-day breakfast consumption, body mass index, physical activity and cardiorespiratory fitness in English schoolchildren. Eur. J. Clin. Nutr..

[45] Thivel D., Aucouturier J., Isacco L., Lazaar N., Ratel S., Doré E., Meyer M., Duché P. (2013). Are eating habits associated with physical fitness in primary school children?. Eat. Behav..

[46] Kohri T., Kaba N., Itoh T., Sasaki S. (2016). Effects of the national school lunch program on bone growth in Japanese elementary school children. J. Nutr. Sci. Vitam..

[47] Liu A.L., Duan Y.F., Hu X.Q., Zou S.R., Qin A.P., Ma G.S. (2011). Change in snacking behaviors of children in four cities of China over 10 years. Chin. J. Sch. Health.

[48] Hu X.Q., Fan Y.O., Hao L.N., Fan J.W., Pan S.X., Ma S.G. (2010). Survey of breakfast behaviors among primary and secondary students in seven cities of China. Acta Nutr. Sin..

[49] Tambalis K.D., Panagiotakos D.B., Psarra G., Sidossis L.S. (2019). Association of cardiorespiratory fitness levels with dietary habits and lifestyle factors in schoolchildren. Appl. Physiol. Nutr. Metab..

[50] Luo D.M., Xu R.B., Hu P.J., Dong B., Zhang B., Song Y., Ma J. (2018). Analysis on the current situation of insumcient sleep and its association with physical exercise among Chinese Han students aged 9–18 years, in 2014. Chin. J. Epidemiol..

[51] Ochiai H., Shirasawa T., Shimada N., Ohtsu T., Nishimura R., Morimoto A., Hoshino H., Tajima N., Kokaze A. (2012). Sleep duration and overweight among elementary schoolchildren: A population-based study in Japan. Acta Med. Okayama.

